# Dosimetric advantages of dual-layer MLC in hypofractionated stereotactic radiotherapy for multiple brain metastases: a comparative study with single-layer MLC

**DOI:** 10.3389/fonc.2025.1564801

**Published:** 2025-05-30

**Authors:** Changyou Zhong, Suyan Bi, Haidong Yu, Zhendong Jiang, Zhitao Dai

**Affiliations:** ^1^ Radiotherapy Department of Meizhou People’s Hospital (Huangtang Hospital), Meizhou, China; ^2^ School of Medical Sciences, Universiti Sains Malaysia, Kelantan, Malaysia; ^3^ National Cancer Center/National Clinical Research Center for Cancer/Cancer Hospital and Shenzhen Hospital, Chinese Academy of Medical Sciences and Peking Union Medical College, Shenzhen, China

**Keywords:** stereotactic radiotherapy, multiple brain metastases, dual-layer MLC, non-coplanar radiotherapy, Halcyon 3.0

## Abstract

**Purpose:**

This study aims to evaluate the dosimetric impact of dual-layer-stacked multi-leaf collimators (MLCs) by comparing stereotactic radiotherapy (SRT) plans for multiple brain metastases (BMs) using the dual-layer MLC of the Halcyon 3.0 system against the single-layer MLC of the Versa HD system.

**Methods:**

Eighteen patients with multiple BMs were retrospectively selected for this study. For each patient, five SRT plans were generated: two using the dual-layer MLC (Halcyon 3.0) and three using the single-layer MLC (Versa HD, comprising two coplanar and one non-coplanar plan). All plans were optimized under identical conditions, with dose normalization ensuring 95% of the target volume received 100% of the prescribed dose. Dosimetric parameters such as D_2%_, D_x_ (the dose of x percent volume), D_98%_, D_mean_, conformity index (CI), homogeneity index (HI), and gradient index (GI) for the planning target volume, as well as D_max_, D_mean,_ and volume-specific doses (V_X_) for organs at risks (OARs), were analyzed. Additionally, treatment time, monitor units (MUs), and radiobiological indices, including equivalent uniform dose (EUD), tumor control probability (TCP), and normal tissue complication probability (NTCP), were assessed.

**Results:**

The Halcyon 3.0 plans with dual-layer MLC demonstrated superior dosimetric outcomes compared to Versa HD coplanar plans, particularly in reducing V_5_, V_10_, V_20_, V_24,_ and D_mean_ for normal brain tissue. The GI of Halcyon 3.0 plans was comparable to that of Versa HD noncoplanar plans. Furthermore, Halcyon 3.0 plans achieved lower EUD and NTCP values for both brainstem and normal brain tissue, matching the performance of Versa HD non-coplanar plans. These findings highlight the efficacy of dual-layer MLC in achieving dosimetric results similar to non-coplanar techniques while offering enhanced protection to OARs.

**Conclusion:**

The dual-layer stacked MLC of the Halcyon 3.0 system provides comparable OAR sparing and dose gradients to non-coplanar Versa HD plans in single-isocenter hypofractionated SRT for multiple BMs, with significant improvements over coplanar Versa HD plans. This suggests that Halcyon 3.0 could be a preferred option for such treatments when non-coplanar setups are not feasible.

## 1 Introduction

Brain metastases (BMs) represent one of the most prevalent intracranial malignancies, affecting 20%–40% of cancer patients (1), with approximately 70% presenting with multiple lesions at diagnosis (2). While whole-brain radiation therapy (WBRT) has historically been a cornerstone for preventing disease progression, stereotactic radiosurgery (SRS) has emerged as a preferred modality due to its superior preservation of neurocognitive function, minimizing quality-of-life compromise in survivors (3–5). With technological advancements, hypofractionated stereotactic radiotherapy (SRT)—administered over 2–5 fractions—has gained clinical traction as a versatile alternative for treating primary and metastatic brain tumors, balancing biological efficacy with logistical feasibility (6).

The management of multiple BMs demands precision in dose delivery to target lesions while sparing critical normal tissues, a challenge addressed by various radiotherapy platforms (7). Systems like the CyberKnife and Gamma Knife offer high conformality but are limited by tumor geometry and accessibility, prompting widespread reliance on linac-based solutions. In contrast, linac-based platforms, which are commonly employed in institutions worldwide, provide practical insights applicable to similar clinical settings. The absence of restrictions on tumor size and location, along with the capability to perform SRS or SRT, makes these platforms the preferred choice for many cancer centers.

**Figure 1:**
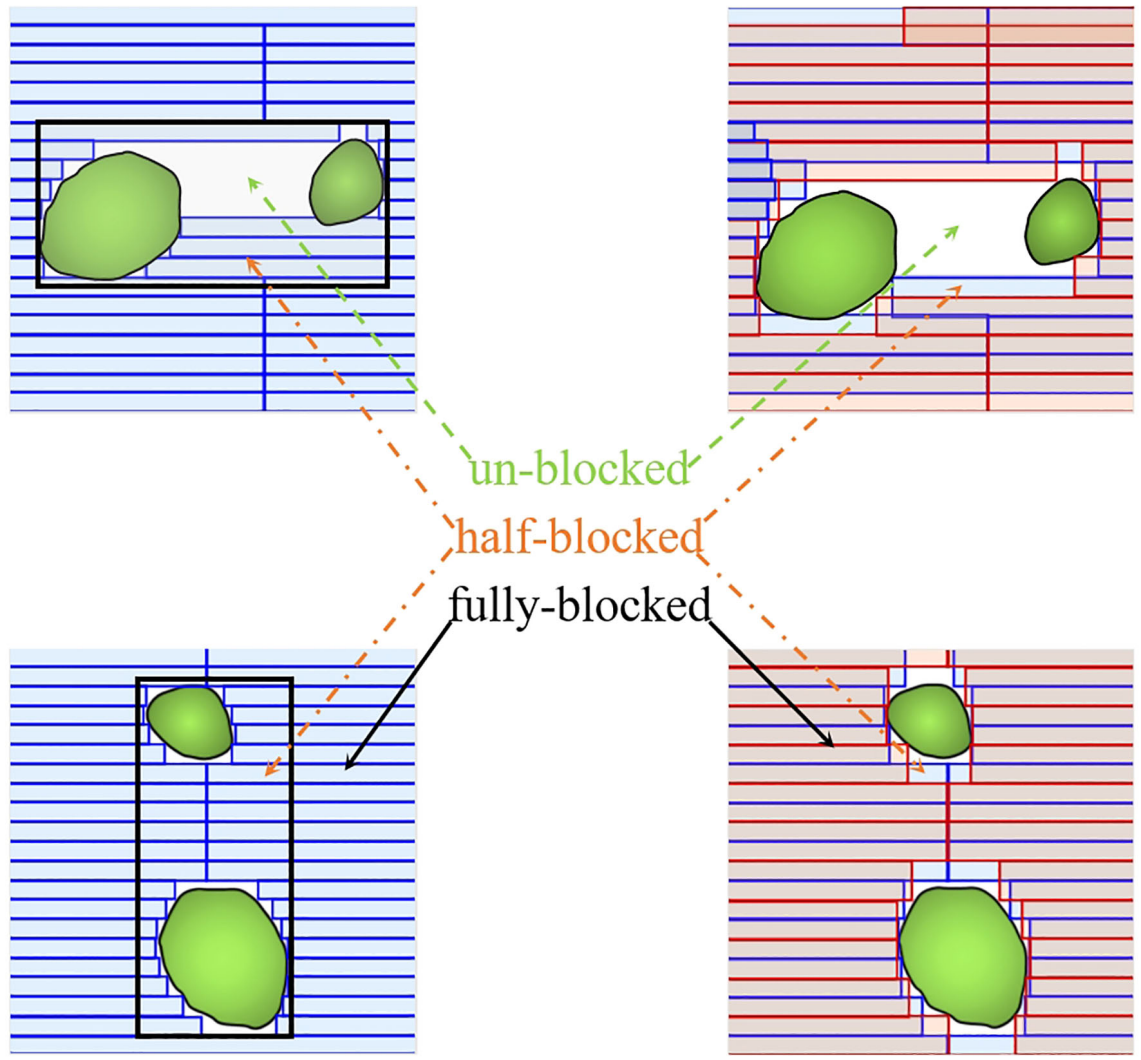
Comparison of the multi-leaf collimator (MLC) designs between the dual-layer Halcyon MLC (red) and the single-layer Versa HD MLC (blue). The dual-layer design of Halcyon MLC reduces leaf-to-leaf leakage and improves dose consistency, as shown by the overlapping areas.

As one of the latest linear accelerator systems, the Halcyon 3.0 (Varian Medical Systems, Palo Alto, CA, USA) features a unique staggered dual-layer stacked multi-leaf collimator (MLC). This system comprises two MLCs, each with a width of 1 cm and an effective resolution of 0.5 cm at the isocenter. It can achieve a maximum MLC speed of 5.0 cm/s while maintaining an extremely low MLC transmission rate of only 0.5% of the primary beam (8, 9). In contrast, the Versa HD system (Elekta Oncology Systems, Crawley, UK) features a single-layer stacked multi-leaf collimator (MLC) with 80 pairs of leaves, each 5 mm wide at the isocenter. This system can achieve a maximum MLC speed of 6.5 cm/s and is capable of delivering flattening filter-free (FFF) photon beams using agility (10). **Figure 1** illustrates some physical design and functionality differences between Halcyon 3.0 MLC and Versa HD MLC. Both devices can be used for SRT treatment based on their excellent dose accuracy control performance. However, the dosimetric difference of SRT plans for multiple BMs based on these two devices has been rarely studied so far.

In this study, we designed the SRT plans for multiple BM cases using the eclipse treatment planning system (TPS) based on the Halcyon (double-layer MLC, DLM) machine mode and then compared these plans with those using the Monaco TPS based on the Elekta Versa HD Agility (single-layer MLC, SLM) machine model.

## 2 Materials and methods

### 2.1 Patient selection

A retrospective analysis was conducted on eighteen patients with multiple BMs (2–10 lesions) treated between May 2020 and April 2023. Prior to treatment, computed tomography (CT) images were acquired on a Discovery RT590CT BigBore (GE Healthcare, Milwaukee, WI) with parameters set as 120 kV, 250 mAs, and 2.5 mm slice thickness. Patients were positioned supine and immobilized using a thermoplastic head mask to ensure reproducible setup. Gross tumor volumes (GTVs) were delineated on fused CT and magnetic resonance imaging (MRI) by two experienced radiation oncologists, incorporating all clinical information. The planning target volume (PTV) was generated by adding a 3-mm isotropic margin to the GTV to account for interfractional setup uncertainties. All contours were reviewed by a senior radiation oncologist to maintain consistency. Patient and target characteristics, including the number of lesions (2–10 per patient) and PTV volume (mean: 25.1 cc; range: 7.9–77.3 cc), are detailed in **Table 1**. The study was approved by the Ethics Committee of Meizhou People’s Hospital (IRB2023-C39), and written informed consent was waived due to its retrospective nature.

**Table 1:** Patient characteristics.

Patient ID	No. of lesions	Volume of each lesion (cc)										Total volumes (cc)	Maximum distance between lesions (cm)		
		A	B	C	D	E	F	G	H	I	J		X	Y	Z
1	2	5.0	2.9									7.9	4.11	5.85	4.75
3	2	4.9	6.2									11.1	9.3	7.0	5.3
3	2	20.5	39.1									59.6	4.51	6.95	5.25
4	3	6.9	7.9	14.9								29.6	5.76	2.72	5.75
5	3	5.8	10.8	13.8								30.6	5.13	6.14	8.01
6	3	11.1	12.1	7.7								30.8	12.18	12.39	4.25
7	3	12.1	18.1	7.2								37.5	5.46	7.51	6.01
8	3	6.5	20.5	23.8								50.9	4.34	5.54	3.25
9	3	34.2	21.5	14.7								70.4	5.42	5.84	6.3
10	3	15.1	9.5	52.8								77.3	6.19	3.54	3.75
11	4	5.5	3.1	5.1	6.6							20.3	6.53	8.29	5.51
12	4	9.8	5.8	10.8	14.2							40.6	5.24	3.92	5.21
13	4	20.1	6.4	10.1	16.0							52.7	7.03	6.09	3.26
14	4	10.1	15.5	9.7	19.9							55.2	8.14	6.09	5.76
15	5	5.6	5.6	5.2	4.8	7.6						28.8	7.92	5.79	6.17
16	5	10.4	3.8	5.9	5.6	4.5						30.1	4.21	8.98	4.58
17	8	5.8	5.5	6.1	3.5	4.6	8.1	3.5	5.9			42.9	4.59	5.32	3.42
18	10	6.9	6.5	7.5	8.4	15.5	5.5	2.7	3.5	6.8	8.7	71.9	4.0	8.7	9.7

### 2.2 Treatment planning

For each patient, five treatment plans were developed: two using the Halcyon 3.0 platform (Varian Medical Systems, Palo Alto, CA, USA) with dual-layer stacked multi-leaf collimators (DLM) and three using the Versa HD platform (Elekta Oncology Systems, Crawley, UK) with single-layer stacked multi-leaf collimators (SLM). The Halcyon 3.0 plans included two coplanar 2-arc VMAT plans: DLM_3/357_ (360°arcs at 0°couch rotation, collimator angles 3°and 357°) and DLM_3/90_ (360°arcs at 0°couch rotation, collimator angles 3°and 90°). Dose calculations were performed using the analytical anisotropic algorithm (AAA) with 1-mm grid spacing, incorporating heterogeneity corrections.

The Versa HD plans comprised two coplanar 2-arc VMAT plans—SLM_3/357_ (collimator angles 3°and 357°) and SLM_3/90_ (collimator angles 3°and 90°), as well as one non-coplanar 4-arc VMAT plan, SLM_nc_ (1 full 360°arc at 0°couch rotation and three 150°partial arcs at couch rotations of 45°, 90°, and 315°). These plans were optimized on the Monaco TPS using the Monte Carlo algorithm with a 1 mm grid. All plans utilized 6 MV FFF beams. Isocenters were automatically placed at the center of mass of the combined PTVs. The prescription dose was 30 Gy in 5 fractions, normalized to cover 95% of the PTV in each plan to ensure consistent target coverage.

Notably, all plans were designed by system-specific dosimetrists in a blinded manner, adhering to identical planning objectives and optimization strategies. The strategy prioritized steep dose fall-off over maximum dose constraints, with plan optimization terminated when further improvements in dose gradient were unattainable (4). This approach was uniformly applied to both platforms to isolate the effect of MLC design on dosimetric outcomes.

### 2.3 Plan comparison

Dose-volume histogram (DVH) data for PTVs and OARs were extracted for analysis. Target evaluation included near-maximum dose(D_2%_), near-minimum dose (D_98%_), and the mean dose(D_mean_) of PTV, along with conformity index (CI, Equation 1), homogeneity index (HI, Equation 2), and gradient index (GI, Equation 3), which were utilized to evaluate the plan quality. CI, HI, and GI were calculated as follows (11, 12).


(1) $$CI=\frac{V_{\text{ref}}}{V_{T}}$$

(2) $$HI=\frac{D_{2\%}-D_{98\%}}{D_{p}}$$

(3) $$GI=\frac{V_{50\%}}{V_{100\%}}$$

where *V*_ref_ is the volume of PTV covered by reference isodose, *V_T_* is the PTV volume, and *D_p_* is the prescription dose. *V*_50%_ and *V*_100%_ are volumes covered by 50% and 100% of prescription dose, respectively.

For OARs, parameters included maximum dose (D_max_), mean dose (D_mean_), and dose-volume metrics (V_5_, V_10_, V_20_, V_24_) for normal brain tissue were recorded, with V_20_ and V_24_ highlighted as critical indicators of radiation-induced necrosis in hypofractionated SRT (6, 13). Brainstem, optic nerves, and optic chiasm were evaluated using structure-specific dose constraints referenced from the TG101 report (14) and Timmerman criteria (15).

### 2.4 TCP and NTCP evaluation

The evaluation of biological effects involved the equivalent uniform dose (EUD, Equation 4) and normal tissue complication probability (NTCP, Equation 5), calculated using Niemierko’s model (16, 17):


(4) $$EUD={\left(\sum_{i}v_{i}d_{i}^{a}\right)}^{1/a}$$

(5) $$NTCP=1/[1+{\left(\frac{TD_{50/5}}{EUD}\right)}^{4\gamma_{50}}]$$

where *v_i_* is the percentage of voxels receiving dose *di*. The *v_i_* and *di* values are acquired from the DVHs, and the sum of *v_i_* over all voxels equals 1. a is a parameter that reflects the dose-response property of distinct organs, and in some literature, the parameter *n* is used with *a* = 1*/n* (18). *TD*_50_ *_/_*_5_ is the dose for achieving a 50% probability of normal tissue complication in 5 years as the OAR is irradiated homogeneously, and *γ*_50_ is the slope of the sigmoidal dose response curve of the OAR. According to reference (17), for brainstem *a* = 7, *TD*_50_ *_/_*_5_ = 65 Gy, *γ*_50_ = 3; for normal brain, *a* = 5, *TD*_50_ *_/_*_5_ = 60 Gy, *γ*_50_ = 3.

### 2.5 Statistical analysis

Data were analyzed using SPSS 22.0 (SPSS Inc., Chicago, IL). Given the non-normal distribution of dosimetric parameters, paired comparisons between plans were performed using the Wilcoxon signed-rank test. A significance threshold of *P <* 0.05 was applied for all statistical tests.

## 3 Results

**Figure 2:**
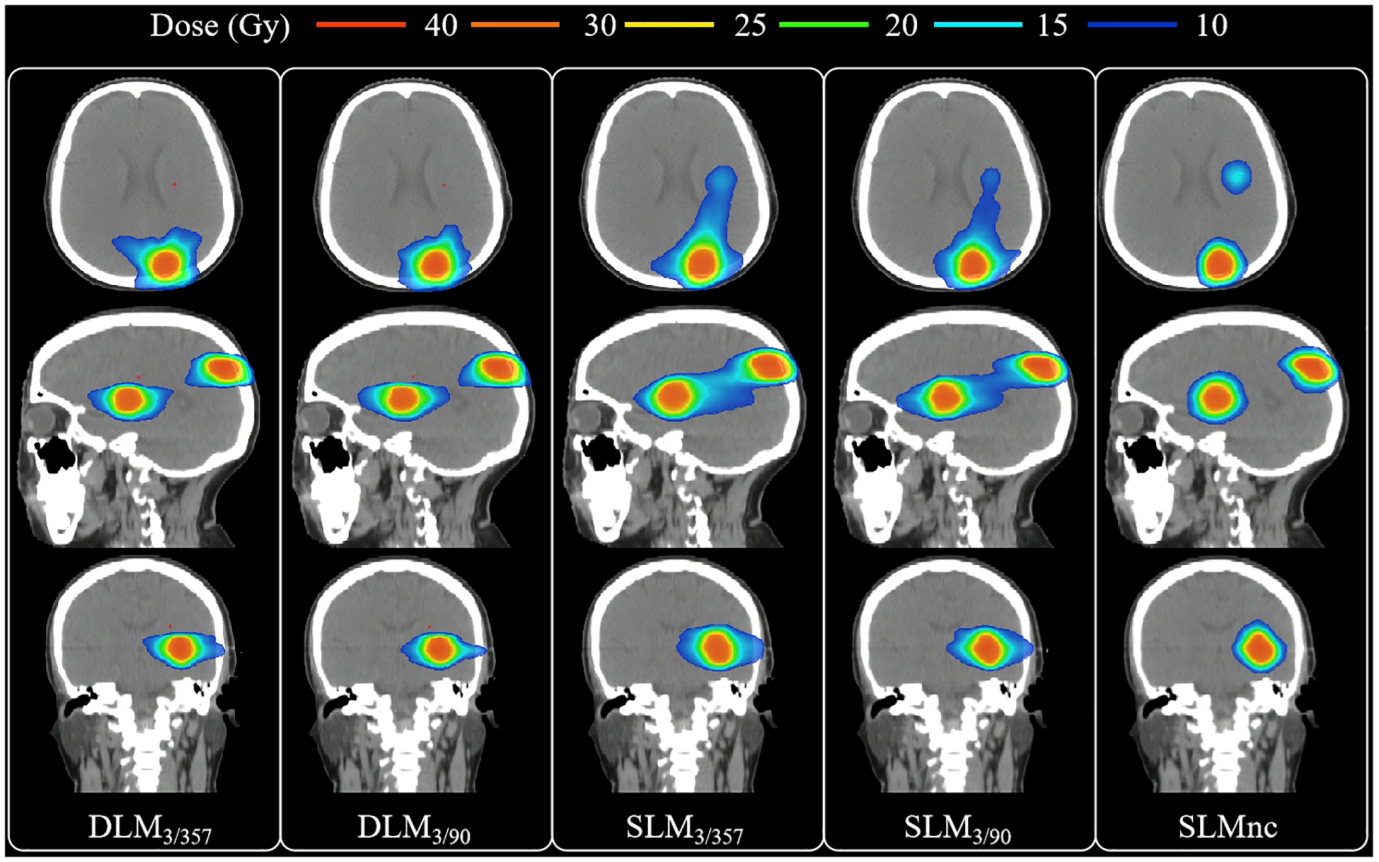
Comparison of 2D dose distribution among five plans for one representative case.

**Figure 3:**
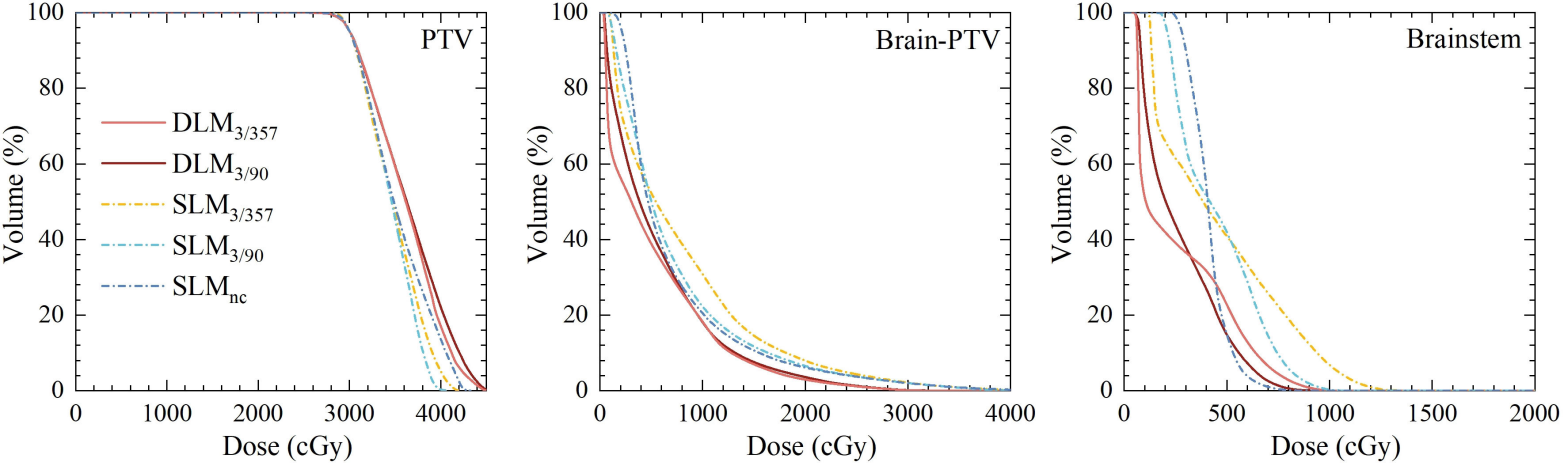
Comparison of dose volume histograms for PTV and OARs between five plans for the representative case.

A total of 90 treatment plans from 18 patients were evaluated, all of which met clinical requirements with adequate target coverage and safe organ doses. Dosimetric parameters were summarized in **Tables 2** and **3**, with representative two-dimensional (2D) dose distributions and DVHs illustrated in **Figures 2** and **3**. Notably, the 10 Gy isodose line of Halcyon 3.0 plans was more conformal to the PTV contour compared to Versa HD plans, indicating reduced low-dose exposure to surrounding tissues.

**Table 2:** Averaged values of dose-volumetric parameters for target.

Metrics	DLM_3/357_ (mean ± SD)	DLM3/90 (mean ± SD)	SLM_3/357_ (mean ± SD)	SLM3/90 (mean ± SD)	SLM_nc_ (mean ± SD)
Coverage (%)	95.1	95.1	95.1	95.1	95.1
D_max_ (cGy)	4118.1 ± 170.9	4126.1 ± 162.6	4069.7 ± 133.6	4100.5 ± 243.3	4081.2 ± 152.7
D_mean_ (cGy)	3495.8 ± 69.8	3515.3 ± 81.5	3447.3 ± 58.5	3431.34 ± 66.1	3475.6 ± 56.9
V100% (cc)	40.9 ± 19.4	40.8 ± 19.5	44.1 ± 19.9	44.1 ± 20.2	43.5 ± 19.9
V50% (cc)	162.9 ± 79.6	165.7 ± 87.8	219.8 ± 142.3	209.4 ± 120.3	161.5 ± 70.9
D_2%_ (cGy)	3964.7 ± 154.1	3976.1 ± 152.3	3865.8 ± 181.5	3887.3 ± 211.2	3879.9 ± 167.5
D_50%_(cGy)	3479.7 ± 65.2	3499.7 ± 76.3	3449.5 ± 61.3	3442.9 ± 67.1	3479.9 ± 52.6
D_98%_ (cGy)	2929.8 ± 18.4	2932.9 ± 22.4	2906.1 ± 76.9	2916.9 ± 15.8	2911.6 ± 21.5
CI	0.833 ± 0.0648	0.836 ± 0.0562	0.8515 ± 0.0535	0.849 ± 0.0667	0.861 ± 0.0510
HI	0.345 ± 0.050	0.348 ± 0.0492	0.3199 ± 0.0669	0.324 ± 0.0718	0.323 ± 0.0545
GI	4.155 ± 0.986	4.216 ± 1.0818	4.991 ± 1.601	4.858 ± 1.339	3.871 ± 0.901
MU	2780.3 ± 513.7	27849.8 ± 464.3	2581.5 ± 641.5	2425.6 ± 616.1	3238.3 ± 672.2
Beam on time (min)	3.6 ± 0.9	3.6 ± 0.7	2.5 ± 0.8	2.2 ± 0.5	3.2 ± 0.8

D_max_ = D0.035cc.

**Table 3:** Averaged values of dose-volumetric parameters for OARs.

Structure	Metrics	DLM_3/357_ (mean ± SD)	DLM_3/90_ (mean ± SD)	SLM_3/357_ (mean ± SD)	SLM_3/90_ (mean ± SD)	SLM_nc_ (mean ± SD)
Brain-PTVs	D_max_ (cGy)	3492.6 ± 151.8	3554.9 ± 197.9	3769.4 ± 672.8	3628.5 ± 205.9	3617.4 ± 171.5
	D_mean_ (cGy)	610.9 ± 166.5	624.6 ± 170.3	762.9 ± 224.4	753.7 ± 226.4	716.9 ± 200.9
	V_5_ Gy (cc)	619.4 ± 191.8	645.0 ± 212.5	745.8 ± 201.8	714.8 ± 189.7	692.1 ± 254.3
	V_10_Gy (cc)	278.4 ± 134.4	290.5 ± 153.7	374.8 ± 186.5	352.0 ± 169.8	277.4 ± 159.9
	V_20_ Gy (cc)	57.1 ± 31.1	55.3 ± 30.1	70.7 ± 31.3	71.3 ± 32.2	61.0 ± 24.4
	V_24_ Gy (cc)	27.8 ± 12.4	27.1 ± 11.5	36.4 ± 13.7	35.8 ± 15.7	22.4 ± 12.6
Brainstem	D_max_ (cGy)	1258.3 ± 692.3	1274.8 ± 744.2	1525.8 ± 782.4	1503.3 ± 692.9	1327.4 ± 655.8
	D_mean_(cGy)	556.1 ± 361.6	551.1 ± 351.8	732.8 ± 63.1	704.1 ± 454.8	687.7 ± 387.3
Optical nerve L	D_max_ (cGy)	397.9 ± 301.0	407.5 ± 266.1	493.8 I 339.1	513.0 ± 445.8	577.4 ± 359.6
	D_mean_ (cGy)	317.8 ± 245.6	356.1 ± 217.6	378.1 ± 242.7	384.3 ± 197.6	439.4 ± 198.8
Optical nerve R	D_max_ (cGy)	397.8 ± 321.5	414.9 ± 310.5	543.5 ± 293.9	449.1 ± 311.6	472.8 ± 228.2
	D_mean_ (cGy)	300.8 ± 268.8	356.7 ± 285.2	470.5 I 331.8	391.5 ± 231.3	384.2 ± 181.6
Optic chiasma	D_max_ (cGy)	536.3 ± 389.2	566.7 ± 342.2	771.5 ± 595.4	793.5 ± 584.3	788.6 ± 478.2
	D_mean_ (cGy)	392.5 ± 292.6	448.5 ± 267.6	588.6 ± 360.7	624.9 ± 404.9	558.5 ± 269.5

### 3.1 Target volume (PTV) evaluation

**Figure 4:**
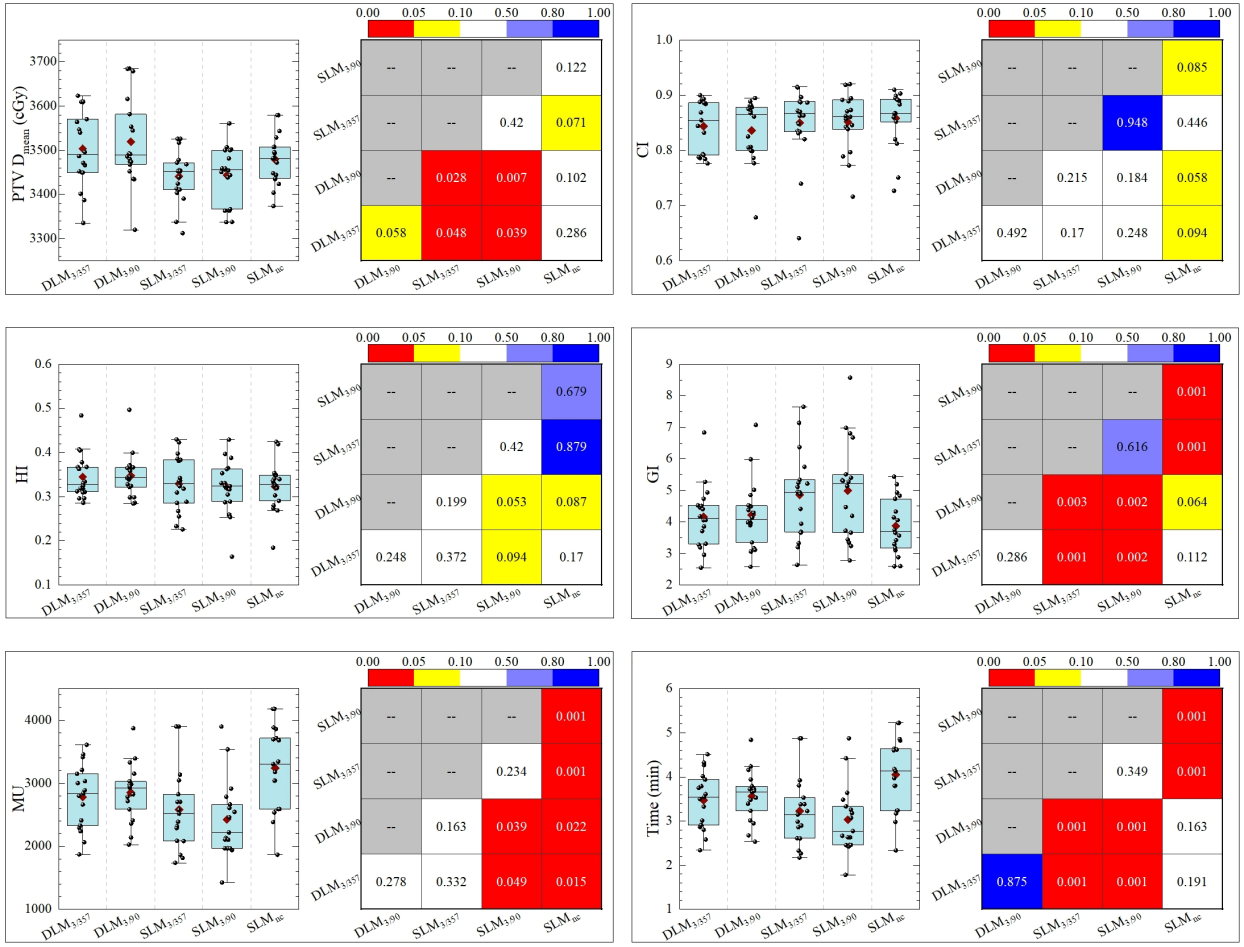
The statistical comparison of key parameters of target between five plans.

Double-layer MLC (DLM, Halcyon 3.0) plans exhibited comparable homogeneity index (HI) and CI to single-layer MLC (SLM, Versa HD) plans, with no significant differences across all five plans, as shown in **Figure 4**. The gradient index (GI), a measure of dose fall-off, was as follows: DLM_3/357_: 4.155 ± 0.986; DLM_3/90_: 4.216 ± 1.0818; SLM_3/357_: 4.991 ± 1.601; SLM_3/90_: 4.858 ± 1.339; and SLM_nc_: 3.871 ± 0.901. The DLM plans achieved a significantly smaller GI compared to coplanar plans of SLM DLM_3/357_ vs. SLM_3/357_: *P <* 0.001; DLM_3/90_ vs. SLM_3/90_: *P* = 0.002), indicating steeper dose gradients. However, GI values for DL-MLC plans were statistically comparable to the non-coplanar plan (DLM_3/357_ vs. SLM_nc_: *P* = 0.112; DLM_3/90_ vs. SLM_nc_: *P* = 0.064), demonstrating equivalent dose gradient quality to non-coplanar techniques. Mean dose (D_mean_) for PTV was slightly higher in DLM plans than in coplanar SLM plans (DLM_3/357_ vs. SLM_3/357_: *P* = 0.048; DLM_3/90_ vs. SLM_3/90_: *P* = 0.007), but closely matched the non-coplanar (DLM_3/357_ vs. SLM_nc_: *P* = 0.286; DLM_3/90_ vs. SLM_nc_: *P* = 0.102). The results for the five plans are DLM_3/357_: 3495.8 ± 69.8 cGy; DLM_3/90_: 3515.3 ± 81.5 cGy; SLM_3/90_: 3431.34 ± 66.1 cGy; SLM_3/357_: 3447.3 ± 58.5 cGy; and SLM_nc_: 3475.6 ± 56.9 cGy, as detailed in **Table 2**.

### 3.2 Treatment efficiency (MU and delivery time)

As depicted in **Table 2** and **Figure 4**, non-coplanar *SLM_nc_* plans required significantly higher monitor units (MUs) than coplanar plans, reflecting the complexity of multi-arc non-coplanar delivery. Halcyon 3.0 plans had longer treatment times compared to Versa HD coplanar plans, primarily due to the lower maximum dose rate of the Halcyon platform (800 MU/min vs. 1,400 MU/min for Versa HD), though this difference did not impact clinical feasibility.

### 3.3 Organ-at-risk sparing

**Figure 5:**
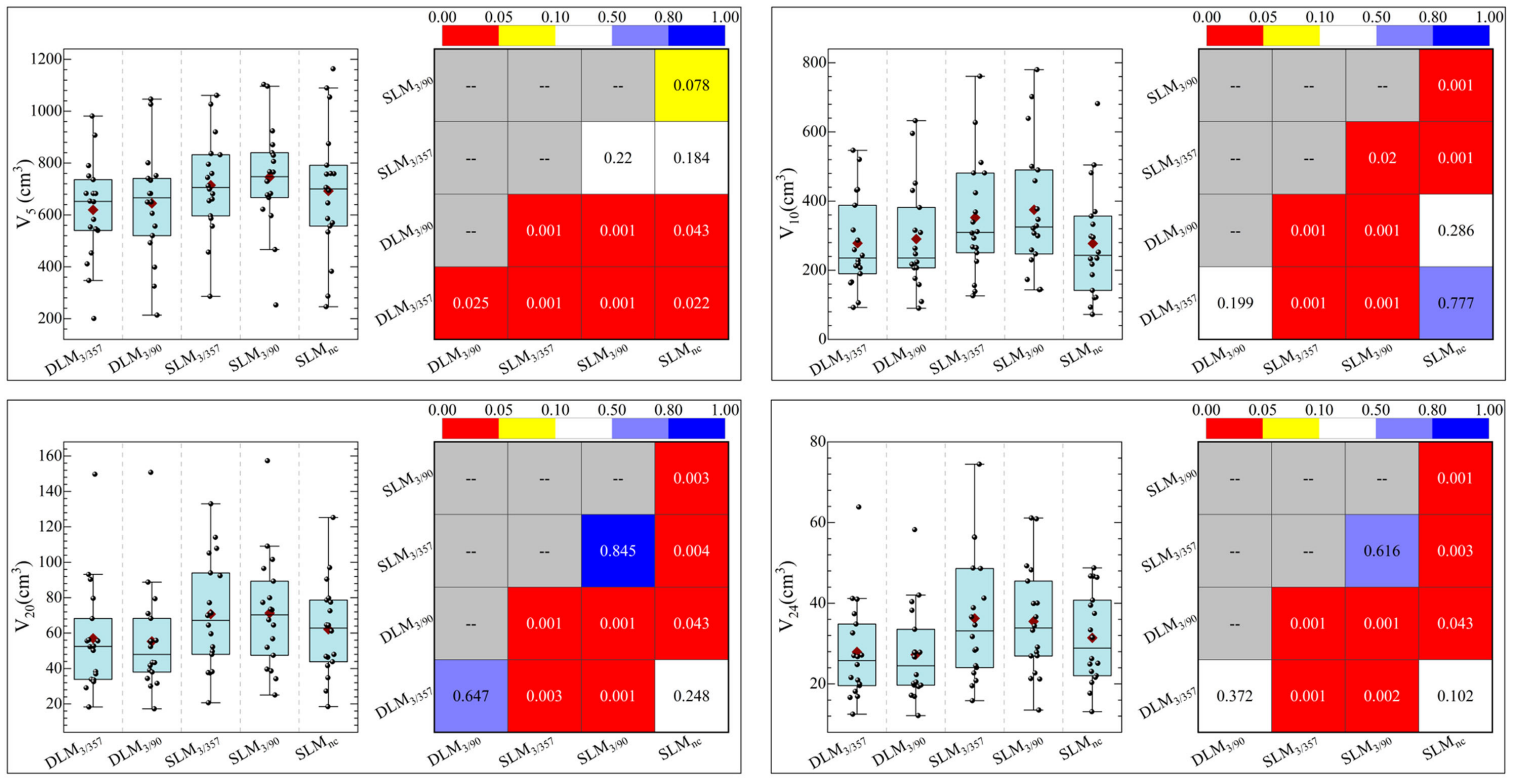
The statistical comparison of key parameters of normal brain between five plans.

The statistical comparison of normal brain tissue was shown in **Figure 5**. DLM plans achieved superior sparing compared to all SLM coplanar plans with significantly lower mean dose (D_mean_) for Brain-PTV compared to all other SLM groups (*P <* 0.001). The DLM_3/357_ plan had the lowest D_mean_ in the BrainPTV, with a value of 610.9± 166.5 cGy, followed by the DLM_3/90_, SLM_nc_, SLM_3/357_, and SLM_3/90_ with the values of 624.6± 170.3 cGy, 716.9± 200.9 cGy, 762.9± 224.4 cGy, and 753.7± 226.4 cGy, respectively. The values of V_20_ (cc) and V_24_ (cc) are particularly noteworthy due to their correlation with radionecrosis following SRT treatment. Our results indicate that DLM plans achieved superior V_20_(cc) and V_24_(cc) values compared to coplanar SLM plans, and were comparable to non-coplanar SLM plans. Additionally, the results for V_10_ (cc) were consistent with these findings. In addition, the low-dose V_5_ (cc) comparison indicated that DLM offered a reduced low-dose volume to normal brain tissue compared to both coplanar (*P <* 0.001) and non-coplanar plans (DLM_3/357_ vs. SLM_nc_: *P* = 0.02, DLM_3/90_ vs. SLM_nc_: *P* = 0.043).

For normal brain tissue (Brain-PTV), DLM plans achieved superior sparing compared to all SLM coplanar plans, with significantly lower mean dose (D_mean_), V_5_, V_10_, V_20_, and V_24_ values (all *P <* 0.001, as shown in **Figure 5** and **Table 3**). Specifically, *DLM*_3_ *_/_*_357_ and *DLM*_3_ *_/_*_90_ plans reduced V_20_ by 19.2% and 22.5%, respectively, compared to *SLM*_3_ *_/_*_357_ and *SLM*_3_ *_/_*_90_. While *SLM_nc_* plans showed comparable *V*_20_ and *V*_24_ to DLM plans, their *V*_5_ values were higher (*P <* 0.05), indicating greater low-dose exposure.

In the brainstem, DLM plans demonstrated lower maximum dose (D_max_) and mean dose (D_mean_) than coplanar SLM plans (all *P <* 0.001) and were marginally better than the non-coplanar *SLM_nc_* plan (*P* = 0.001 and *P* = 0.002 for *DLM*_3_ *_/_*_357_ and *DLM*_3_ *_/_*_90_ vs. *SLM_nc_*, respectively), highlighting improved protection of this critical structure. Optic nerves and chiasma received low doses across all plans, with no significant differences between platforms (all *P >* 0.05, as shown in **Table 3**).

### 3.4 Radiobiological parameters for normal brain and brainstem

**Figure 6:**
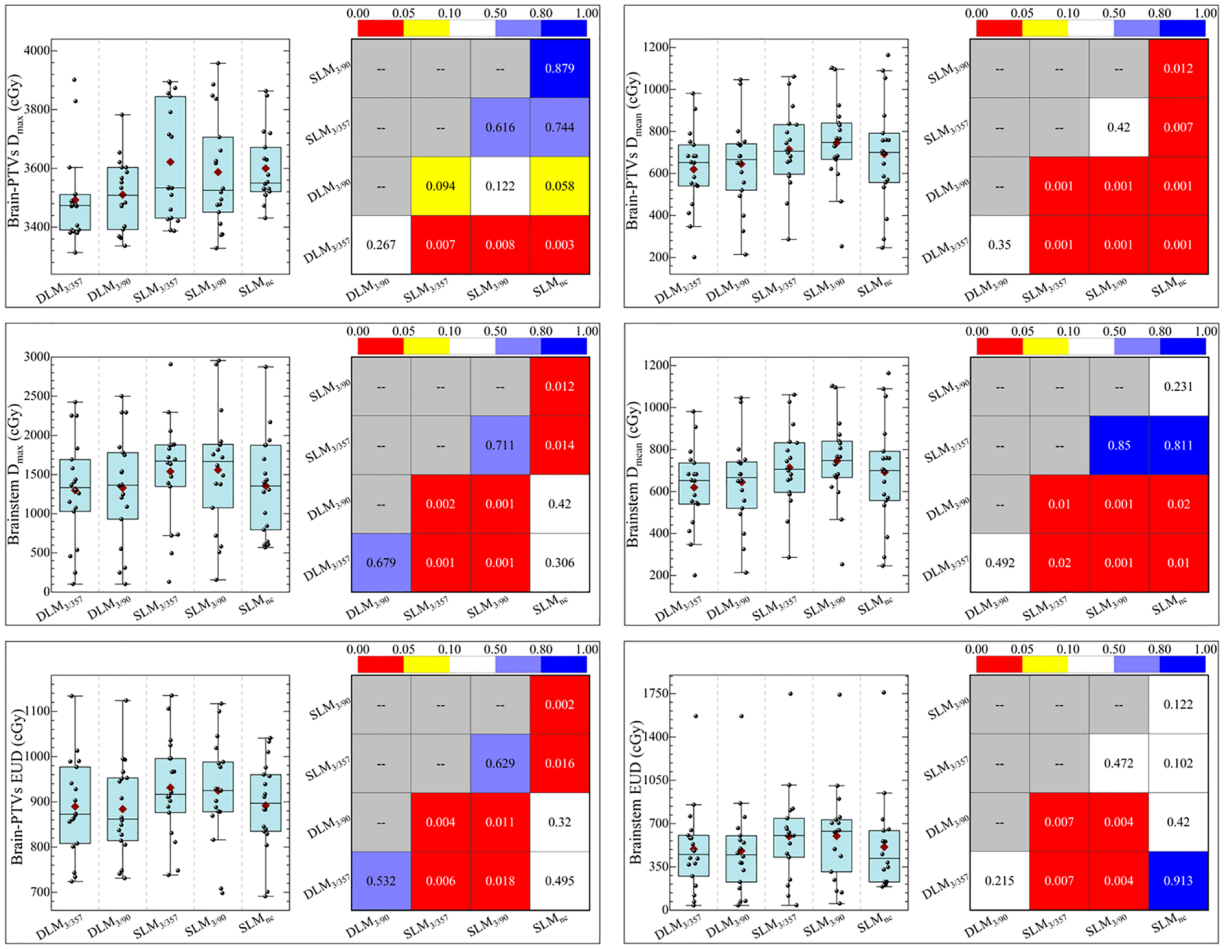
Statistical comparison of D_max_, D_mean_, EUD, and NTCP of brainstem and normal brain between five plans.

DLM plans yielded lower EUD for both normal brain tissue and the brainstem compared to coplanar SLM plans (*P <* 0.05), which correlated with reduced NTCP. Specifically, NTCP values for normal brain tissue were 0.35%–0.13% in DLM plans versus 0.41%–0.14% in coplanar SL-MLC plans (*P <* 0.05), and brainstem NTCP was 0.11%–0.12% in DLM versus 0.16%–0.17% in coplanar SLM plans (*P <* 0.05, as shown in **Table 4** and **Figure 6**). These advantages were maintained when compared to non-coplanar *SLM_nc_* plans, with no significant differences in EUD or NTCP (*P >* 0.05), confirming equivalent radiobiological safety.

**Table 4:** EUD and NTCP of braistem and normal brain.

Structure	Metrics	DLM_3/357_ (mean ± SD)	DLM3/90 (mean ± SD)	SLM_3/357_ (mean ± SD)	SLM3/90 (mean ± SD)	SLM_nc_ (mean ± SD)
Brainstem	EUD (cGy)	494.1 ± 353.2	479.2 ± 359.0	599.4 ± 390.2	597.4 ± 385.2	511.1 ± 379.5
	NTCP (%)	0.11 ± 0.26	0.12 ± 0.27	0.17 ± 0.36	0.16 ± 0.36	0.16 ± 0.38
Brain-PTV	EUD (cGy)	902.1 ± 111.2	898.2 ± 101.2	941.2 ± 112.4	958.4 ± 112.2	912.4 ± 98.2
	NTCP (%)	0.35 ± 0.13	0.35 ± 0.13	0.41 ± 0.13	0.41 ± 0.14	0.36 ± 0.11

## 4 Discussions

In this study, we evaluated the performance of the Halcyon 3.0 platform (double-layer MLC, DLM) and the Versa HD platform (single-layer MLC, SLM) in hypofractionated stereotactic radiation therapy (SRT) for multiple BMs. All five plans across both platforms met clinical criteria, demonstrating adequate target coverage, steep dose gradients, and safe organ-at-risk (OAR) doses. The DLM system demonstrated superior normal tissue sparing, particularly in terms of V_5_(cc) and D _mean_ for normal brain tissue, while achieving dose conformity and gradient comparable to non-coplanar SLM plans. These findings highlight the potential of dual-layer collimation to balance precision and efficiency in metastatic brain radiotherapy.

The advantage of DLM in OAR protection can be attributed to its unique design: the Halcyon 3.0’s dual-layer MLC, featuring 77 mm leaf thickness and staggered interlayer positioning, effectively minimizes beam transmission (¡0.5%) compared to the Versa HD’s single-layer MLC (1.4%–1.5% transmission) (8–10, 19). This reduced leakage is critical during intensity-modulated optimization, as it limits unintended radiation exposure to normal tissues outside the target volume. Consequently, DLM plans showed significantly lower *V*_20_ and *V*_24_—key metrics for radiation-induced necrosis—than coplanar SLM plans and were comparable to non-coplanar SLM plans, which typically require more complex beam arrangements.

Notably, while non-coplanar SLM plans improve dose fall-off within the target plane, they increase low-dose spread in non-target axial planes, as evidenced by higher *V*_5_ values compared to DLM. This trade-off between dose conformity and low-dose volume highlights the clinical dilemma of noncoplanar techniques, which are associated with longer treatment times, potential setup errors from couch rotation (20), and incompatibility with cone-beam CT (CBCT) guidance (21). In contrast, DLM achieves steep dose gradients in coplanar arcs, offering a practical alternative when non-coplanar delivery is contraindicated due to logistical or technical constraints.

A critical challenge in multi-target SRT is the “island blocking” phenomenon, where overlapping MLC leaf usage between distant lesions exposes intervening brain tissue. Although collimator angles of 3°/357° and 3°/90° were tested to mitigate this issue, no significant differences were observed between these configurations, possibly due to heterogeneous lesion distributions. Future studies could explore subarc collimator angle optimization (SACAO), which dynamically adjusts leaf orientation to avoid shared MLC segments, potentially enhancing dose conformity for complex target geometries. The Halcyon 3.0’s lack of non-coplanar arc capability and 6D motion correction introduces setup uncertainty risks, particularly for lesions distant from the isocenter. Rotational errors, even minor ones, can compromise target coverage and OAR safety in single-isocenter plans, as previously reported (22–24). Clinicians using this platform must prioritize rigorous patient immobilization and daily image guidance to mitigate such risks, despite the system’s limitations in addressing rotational mismatches. Particularly, when using single-isocenter VMAT to treat multiple BMs with SRT, attention must be given to managing rotational uncertainties in patient setup (25–27). In multi-target single-isocenter treatment, rotation tolerances are more stringent because targets distant from the isocenter are highly sensitive to residual rotational mismatches between planned and actual treatment positions. Study (28) investigated the connection between rotational setup errors and dosimetric parameters in single-isocentric VMAT SRS for multiple target cases. Their findings indicated that D_95%_ values deteriorated to 60% of the prescribed dose during uniform rotations of 2° around three axes. This study focused solely on the dosimetric effects on the planning target volume (PTV). However, if the rotational errors affecting the GTV were taken into account, the variations could be more pronounced, depending on the extent of the margin used for expansion. Undoubtedly, it can be predicted that when the rotation error reaches a certain degree, it will affect the dose coverage of GTV. Studies (29, 30) have addressed this issue by proposing methods to obtain a non-uniform clinical target volume (CTV) to PTV margin, accounting for both rotational and translational errors. 6D calibration, which couch correction is used to perform rotational corrections around the pitch, roll, and yaw axes, will significantly improve the SRT treatment accuracy (30). However, for reasons, such as safety and treatment complexity, non-coplanar has been constrained by the current machine design—the Halcyon 3.0, which does not support non-coplanar arcs. The daily CBCT online calibration in Halcyon 3.0 does not address rotational errors. Consequently, in single isocenter treatments for multiple BMs, the absence of 6D calibration in Halcyon presents a significant challenge, necessitating greater attention to rotational errors.

This study has several limitations. First, it was limited to plan comparisons without physical delivery validation, necessitating future *in-vivo* dosimetry verification. Second, the absence of direct comparisons with CyberKnife or HyperArc technologies restricts the generalizability of findings, though linac-based platforms remain the focus of most clinical settings. Third, only six MV FFF beams were evaluated, leaving energy-dependent dosimetric differences unaddressed. Last, SACAO was not incorporated into plan optimization, a technique shown to improve multi-target conformity and warranting further investigation.

## 5 Conclusion

Our results clearly indicate that the Halcyon 3.0 planning system, equipped with its dual-layer MLC, performs comparably to non-coplanar Versa HD plans in treating multiple BMs. It effectively delivers lower radiation doses to normal brain tissues and the brainstem compared to the coplanar Versa HD plans. These findings highlight the capability of the dual-layer MLC to enhance dose conformity and minimize exposure to organs at risk (OARs) without requiring complex non-coplanar beam arrangements. Consequently, the Halcyon 3.0 system emerges as a preferred choice for single-isocenter SRT in patients with multiple BMs, particularly in scenarios where non-coplanar setups are impractical or contraindicated. However, the absence of 6D correction capabilities in the Halcyon 3.0 system necessitates meticulous attention to rotational setup errors, underscoring the importance of robust patient immobilization and daily image guidance to ensure treatment precision and safety. Future studies should explore strategies to mitigate setup uncertainties and further validate these findings across larger patient cohorts.

## Data Availability

The datasets presented in this article are not readily available to keep data secure, private, and safe from breaches or damage. Requests to access the datasets should be directed to zcy_mz@sina.com.
